# Wills’ Hospital—Service of Dr. John Neill

**Published:** 1850-11

**Authors:** A. F. Mac Intyre

**Affiliations:** Resident Physician; Wills’ Hospital, Philadelphia


*Wills' Hospital—Service of* Dr. John Neill. *Cases discharged from July 1st to October 1st,* 1850.

| | Cured. | Relieved. | Incurable. |
|---|---|---|---|
| Amaurosis, | 0 | 2 | 0 |
| Closure of the pupil, | 1 | 0 | 1 |
| Amaurosis and cataract, | 0 | 1 | 0 |
| Catarrhal conjunctivitis, | 2 | 0 | 0 |
| Acute " | 3 | 0 | 0 |
| Chronic " | 3 | 0 | 0 |
| Granular " | 2 | 2 | 0 |
| Cataract, | 0 | 1 | 0 |
| Amblyopia, | 0 | 1 | 0 |
| Scrofulous corneitis, | 1 | 0 | 0 |
| Injury of the eye, | 1 | 1 | 0 |
| Staphyloma scleroticæ, | 1 | 0 | 0 |
| Pustular ophthalmia, | 2 | 0 | 0 |
| Inflammation of the orbit, | 1 | 0 | 0 |
| Opthalmia tarsi, | 3 | 0 | 0 |
| Ulcer of the cornea, | 4 | 0 | 0 |
| Iritis, | 1 | 0 | 0 |
| Hypopion, | 0 | 1 | 0 |
| Entropium, | 0 | 1 | 0 |
| | 25 | 10 | 1 |

*Wills' Hospital Dispensary—Cases treated.*

| | | | |
|---|---|---|---|
| Acute conjunctivitis, | 10 | Ulcer of the cornea, | 5 |
| Chronic " | 9 | Granular lids, | 7 |
| Catarrhal " | 11 | Amaurosis, (partial) | 11 |
| Scrofulous " | 13 | Ophthalmia tarsi, | 9 |
| Pustular " | 7 | Tumor of the lid, | 2 |
| Purulent " | 4 | Adhesion of the lid to the globe, | 1 |
| Corneitis, | 3 | Inflammation of lach'l sac, | 2 |
| Iritis, | 6 | | |
| Retinitis, | 1 | Total | 101 |

*Conjunctivitis.*—The chronic, catarrhal, and granular forms of conjunctivitis, were treated chiefly by strong solutions of the nitrate of silver, alternated occasionally as the more active symptoms subsided with the sulphate of copper in substance, and the liquor plumbi sub-acetatis. Under this plan the cases improved satisfactorily.

*Corneitis.*—In the single case discharged cured, the disease occurred in a scrofulous subject, and the treatment was modified accordingly. During the active stage of inflammation, the mild chloride of mercury was cautiously used in connection with the sulphate of quinine. By this combined plan the disease was rapidly subdued, and only a slight opacity remained as the result of the corneitis. Great importance was attached by Dr. Neill to the administration of tonics in connection with mercurials in the treatment of strumous corneitis.

*Photophobia.*—The intolerance of light in cases of strumous ophthalmia, was readily subdued in a large number of cases by the external application of the tincture of iodine, painted freely over the brows, temples, and upper portions of the cheek, with a camel's hair pencil.

*Staphyloma Scleroticæ.*—Michael Faulker, a common laborer, æt. 37, a native of N. Y., having good general health, was admitted to the hospital, July 30, 1850, with obscure disease of the right eye. He applied for relief because of the sympathetic irritation which the diseased eye kept up in the left, or sound one. He came laboring under great anxiety of mind, as he had been told by high surgical authority that the disease was malignant. After carefully examining his diseased eye, Dr. Neill was satisfied that the disease was *not* malignant, and he admitted him with the intention of operating for his relief, by discharging the contents of the globe and allowing it to collapse. At the time of his admission, the globe was so much distended, that the lids would not close over it; the cornea was opake and conoidal, and at the external angle of the eye, the sclerotica was thinned and distended, so as to form a slightly conical tumour, of a leaden or purplish hue. The conjunctiva was in a condition of chronic inflammation. Some years before, he had this eye injured in a stone quarry; inflammation of the eye followed, which in three months time subsided, leaving the eye sightless. He had suffered often since then with attacks of inflammation up to the time he applied for admission to the hospital. Five years ago, the globe began to enlarge, and continued gradually to increase, without causing him pain or any other inconvenience, except the frequent inflammatory attacks when exposed. July 12, Dr. Neill determined to operate; the patient was placed upon the operating table, and with a Beer's knife a section of the cornea was made, as in the operation for the extraction of cataract; the aqueous humour, and the lens, which was opake and partially dissolved, escaped. Behind the position of the lens appeared a dense septum, this was torn away with the curette, and immediately a large quantity of dark colored fluid, seeming to consist of disorganized vitreous humour and blood, flowed from the globe; this fluid continued to flow for half an hour, during which time the patient was left upon the table, with the lids closed over the globe. The blood was probably derived from the rupture of the vessels of the choroid coat, when the great pressure of the fluid which distended the globe was removed. For two days after the operation, the patient seemed to do well, but on the third, the eye became greatly distended, and exceedingly painful. By the use of soothing applications and anodynes, the pain subsided in a few days. During the third week the globe began to diminish in size, and continued gradually to become smaller until August 31, when he was discharged entirely relieved; the globe had resumed its natural shape and size, and the conjunctivitis was completely removed. The history of this case seems to illustrate emphatically the importance of a correct diagnosis in certain forms of ophthalmic disease.

A. F. Mac Intyre, M. D.,

Resident Physician.

*Wills' Hospital, Philadelphia, October*, 1850.

